# Complete genomic characteristics and pathogenic analysis of the newly emerged classical swine fever virus in China

**DOI:** 10.1186/s12917-018-1504-2

**Published:** 2018-06-25

**Authors:** Hongliang Zhang, Chaoliang Leng, Zhijun Tian, Chunxiao Liu, Jiazeng Chen, Yun Bai, Zhen Li, Lirun Xiang, Hongyue Zhai, Qian Wang, Jinmei Peng, Tongqing An, Yunchao Kan, Lunguang Yao, Xufu Yang, Xuehui Cai, Guangzhi Tong

**Affiliations:** 1 State Key Laboratory of Veterinary Biotechnology, Harbin Veterinary Research Institute, Chinese Academy of Agricultural Sciences, Harbin, 150001 China; 2 Henan Key Laboratory of Insect Biology in Funiu Mountain, Henan Provincial Engineering Laboratory of Insects Bio-reactor, China-UK-NYNU-RRes Joint Laboratory of Insect Biology, Nanyang Normal University, Nanyang, 473061 China; 3 Shanghai Veterinary Research Institute, Chinese Academy of Agricultural Sciences, No. 518, Ziyue Road, Minhang District, Shanghai, 200241 China; 4 North Guangdong Collaborative Innovation and Development Center of Pig Farming and Disease Control, Shaoguan University, Shaoguan, 512005 China

**Keywords:** Swine, Classical swine fever virus, Sub-genotype 2.1d, Molecular characteristics, Pathogenicity

## Abstract

**Background:**

Classical swine fever (CSF) is one of the most devastating and highly contagious viral diseases in the world. Since late 2014, outbreaks of a new sub-genotype 2.1d CSF virus (CSFV) had caused substantial economic losses in numbers of C-strain vaccinated swine farms in China. The objective of the present study was to explore the genomic characteristics and pathogenicity of the newly emerged CSFV isolates in China during 2014–2015.

**Results:**

All the new 8 CSFV isolates belonged to genetic sub-genotype 2.1d. Some genomic variations or deletions were found in the UTRs and E2 of these new isolates. In addition, the pathogenicity of HLJ1 was less than Shimen, suggesting the HLJ1 of sub-genotype 2.1d may be a moderated pathogenic isolate and the C-strain vaccine can supply complete protection.

**Conclusions:**

The new CSFV isolates with unique genomic characteristics and moderate pathogenicity can be epidemic in many large-scale C-strain vaccinated swine farms. This study provides the information should be merited special attention on establishing prevention and control policies for CSF.

## Background

Classical swine fever virus (CSFV), the etiological agent of classical swine fever (CSF), is a single-strand, positive-sense RNA virus that belongs to the genus *Pestivirus* of the family *Flaviviridae* [1]. CSFV has a genome of about 12.3 kb comprising a single long open reading frame (ORF) coding for a polyprotein of 3, 898 amino acids flanked by two non-coding regions at the 5′ and 3′ untranslated region (UTR) [2]. The polyprotein undergoes proteolysis to produce four structural proteins (C, E^rns^, E1, and E2) and eight non-structural proteins (N^pro^, P7, NS2, NS3, NS4A, NS4B, NS5A, and NS5B) [3, 4].

Based on E2 and partial NS5B genes, CSFV isolates were originally classified into two genotypes and five sub-genotypes [5]. To date, CSFV have been divided into three groups with three or four subgroups (1.1, 1.2, 1.3; 2.1, 2.2, 2.3; and 3.1, 3.2, 3.3, 3.4) [6]. Sub-genotype 2.1 has been further divided into 2.1a and 2.1b groups [7, 8]. And Gong et al. reported that current CSFV sub-genotype 2.1 virus isolates in the world could be divided into 10 sub-genotypes (2.1a–2.1j) [9]. In mainland China, four sub-genotypes (1.1, 2.1, 2.2, and 2.3) of CSFV were identified, all of which contributed to CSFV outbreaks [10]. Shen et al. reported that subgroup 1.1 CSFV isolates circulated in some distinct region of south China [11]. However, sub-genotype 2.1b has become predominant within the last decade [12–14]. In recent years, sub-genotype 2.1c and 1.4 were defined [15, 16]. Recently, we confirmed a new sub-genotype 2.1d of CSFV [17]. And then the newly emerged CSFV isolates of China in 2014–2015 were also characterized by our laboratory. In the present study, the complete genomes of 8 newly emerged CSFV isolates in China in 2014–2015 were sequenced and analyzed. Moreover, the pathogenicity of one of these isolates, HLJ1, was evaluated.

## Methods

### Clinical samples, virus isolation and virus strains

More than 20 Clinical samples of lung, lymphatic, and spleen tissue were collected from pigs suspected to have CSFV infections in Shandong, Jiangsu, Hebei, Jilin and Heilongjiang provinces of China between October 2014 and August 2015.

Swine testicular (ST) cells were used for CSFV propagation and titration in RPMI Medium 1640 (Gibco, USA) supplemented with 10% heat-inactivated fetal bovine serum (FBS) (Invitrogen), 0.1 mg/ml streptomycin and 100 U/ml antibiotic penicillin. Clinical tissues (Lungs) were homogenized using TissuLyser II (Qiagen, Germany) and centrifuged 10, 000×g for 10 min. The supernatant was filtered through a 0.45-μm filter and transferred to ST cells monolayer. The cells were incubated at 37 °C for 1 h. Then prepared medium was added with 6 ml in volume and the cells were incubated for another 72 h. The cultures were harvested and then stored at − 80 °C as viral stocks. CSFV adaptation in ST cells was conducted for at least 15 passages, and some normally co-infected porcine pathogens, including porcine reproductive and respiratory syndrome virus (PRRSV), pseudorabies virus (PRV), porcine circovirus type 2 (PCV2) and mycoplasma were detected in the viral stocks.

The cultures of the third passage of CSFV HLJ1 in ST cells were quantified and used for challenge virus. The CSFV Shimen strain, a highly virulent strain isolated in China in 1945, were also used as a reference challenge virus in the present study.

### Primer design and RT-PCR

Six pairs of primers based on the published known sequence of CSFV Zj0801 (GenBank accession no. FJ529205) were designed for PCR analysis (Table 1). The cultures were prepared for reverse transcription PCR (RT-PCR) using an RNA extraction QIAamp viral RNA Mini Kit (Qiagen, Germany). RT-PCR was conducted as previously reported [17].

**Table 1:** Primers used for the amplification of the full-length CSFV

Fragment	Primer sequence (5′-3′) ^a^	Position in genome^a^	Product size (bp)^a^
CSFV-A	GTATACGAGGTTAGTTCATTCTCGT GATTACCAGAGAAAGCAACAAGAAT	1-2047	2047
CSFV-B	GATAATAGGCCCCGGTAAATTTGAC TTTCCTTACAGGTCCCTCGCTAGAG	2023–3313	1291
CSFV-C	AAATGAGACGGGTTACAGGGTA CATCCCGTAGATCTCTTCACCTCCA	3124–4771	1648
CSFV-D	CATAGATGAAATAGCTGGCGGGACC TAGTGCTCTGCCAGCCTCCACAGTG	4564–7171	2608
CSFV-E	TCTGCTGATATCAGAGGAGCTG GCTTACCCAGACTTAATGTTTCTAG	6866–9668	2803
CSFV-F	GCCCTATGTAAGGTCGACACCGCTC GGGCCGTTAGGAAATTACCTTAGTC	9572–12,296	2725

^a^The sequence of primer sequence, position in genome and product size with respect to the CSFV Zj0801 (accession no. FJ529205) genome

### Genome cloning and sequencing

Genome cloning and sequencing was conducted as previously reported [17]. Briefly, RT-PCR products were analyzed by using 1% agarose gel electrophoresis, and the target fragments were excised from gels for purification using a Gel Extraction Kit (OMEGA, USA). The purified PCR products were then cloned into a pMD18-T vector (TaKaRa Dalian, China) and sequenced by Comate Bioscience (Jilin, China).

### Phylogenetic analysis

**Fig. 1:**
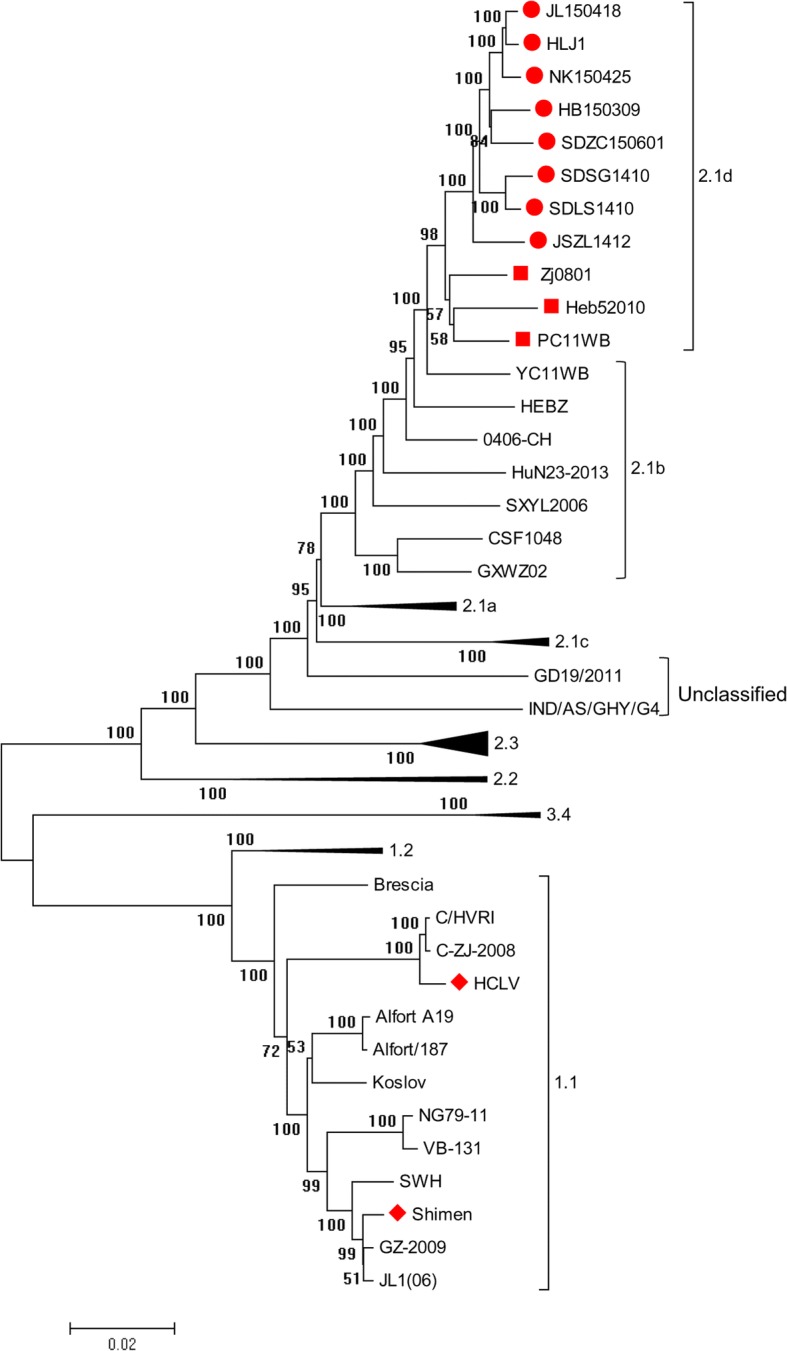
Phylogenetic analysis of the 8 new isolates and 44 reference strains based on the complete CSFV genomes. The 8 new CSFV isolates and 3 reference strains were located in a branch belonging to 2.1d sub-genotype, labeled by  and , respectively. The Shimen strain and its attenuated live vaccine strain, HCLV, were located in another branch belonging to 1.1 sub-genotype, labeled by

The 8 new CSFV isolates and 44 reference CSFV strains (Tables 2 and 3), based on the full of genome, were used for construction of phylogenetic tree (Fig. 1). The phylogenetic tree was constructed using MEGA 5.1 with the maximum likelihood method based on 1000 bootstraps [18]. Multiple sequence alignments were generated using MUSCLE and MEGA software [19].

**Table 2:** Information of 8 newly emerged sub-genotype 2.1d CSFVs

No.	Isolates	Sample	Time	Area
1	SDLS1410	Lung	2014.10	Shandong
2	SDSG1410	Lung	2014.10	Shandong
3	JSZL1412	Serum	2014.12	Jiangsu
4	HB150309	Lung	2015.03	Hebei
5	JL150418	Lung	2015.04	Jilin
6	NK150425	Lung	2015.04	Heilongjiang
7	SDZC150601	Lung	2015.06	Shandong
8	HLJ1	Lung	2015.08	Heilongjiang

**Table 3:** The reference CSFVs used in this study

No.	Virus strain	Year	Origin	Genotype	Accession no.
1	Alfort 187	2010	Switzerland	1.1	X87939
2	Alfort A19	1997	France	1.1	U90951
3	Brescia	1998	Switzerland	1.1	AF091661
4	C-ZJ-2008	2008	China	1.1	HM175885
5	GZ-2009	2009	China	1.1	HQ380231
6	HCLV	1999	China	1.1	AF091507
7	HVRI	2006	China	1.1	AY805221
8	JL1(06)	2006	China	1.1	EU497410
9	Koslov	2010	Germany	1.1	HM237795
10	NG79–11	2014	India	1.1	KC503764
11	Shimen	1999	China	1.1	AF092448
12	VB-131	2014	India	1.1	KM262189
13	Bresciax 90	1990	Netherlands	1.2	M31768
14	RUCSFPLUM	2001	USA	1.2	AY578688
15	CSF0277	1997	Germany	2.1	JQ411566
16	96TD	2005	China	2.1a	AY554397
17	Paderborn	2001	Denmark	2.1a	AY072924
18	SXCDK	2009	China	2.1a	GQ923951
19	0406-CH	2005	China	2.1b	AY568569
20	CSF1048	2009	Germany	2.1b	HQ148063
21	GXWZ02	2003	China	2.1b	AY367767
22	HEBZ	2009	China	2.1b	GU592790
23	HuN23–2013	2013	China	2.1b	KP233071
24	SXYL2006	2006	China	2.1b	GQ122383
25	YC11WB	2011	Korea	2.1b	KC149990
26	GXF29/2013	2013	China	2.1c	KP233070
27	HNLY-2011	2011	China	2.1c	JX262391
28	HNSD-2012	2012	China	2.1c	JX218094
29	Heb52010	2010	China	2.1d	JQ268754
30	HLJZZ2014	2014	China	2.1d	KU375260
31	PC11WB	2011	Korea	2.1d	KC149991
32	Zj0801	2008	China	2.1d	FJ529205
33	39	2001	China	2.2	AF407339
34	LAL-290	2012	India	2.2	KC851953
35	Alfort/Tuebingen	1989	Germany	2.3	J04358
36	Borken	2006	Germany	2.3	GU233731
37	Euskirchen	2005	Germany	2.3	GU233732
38	Hennef	2009	Germany	2.3	GU233733
39	Jambul	2007	Bulgaria	2.3	HQ148062
40	Novska	2002	Croatia	2.3	HQ148061
41	Roesrath	2009	Germany	2.3	GU233734
42	Sp01	2001	Spain	2.3	FJ265020
43	Uelzen	2004	Germany	2.3	GU324242
44	JJ9811	1998	Korea	3.2	KF669877
45	P97	2006	–	3.4	L49347
46	TWN	1994	China	3.4	AY646427

### Nucleotides and amino acid analysis

The complete genome sequences and deduced amino acid sequences of the 8 new isolates were analyzed and their homologies with other 8 representative CSFV strains were compared using the Clustal W method of Lasergene (Version 7.1) (DNASTAR Inc., Madison, WI, USA) (Table 4).

**Table 4:** Detailed comparison of the full-length genomes of the 8 newly emerged sub-genotype 2.1d CSFVs to other CSFV representative isolates (%)

	Shimen (1.1)	Paderborn (2.1a)	HEBZ (2.1b)	HNSD-2012 (2.1c)	Zj0801 (2.1d)	CSFV39 (2.2)	Alfort (2.3)	TWN (3.4)
Nucleotides								
5’UTR	91.4–92.2	96.5–97.3	95.4–96.2	94.4–95.2	97.0–98.7	91.6–92.5	93.3–94.1	90.3–91.9
N^pro^	87.1–88.1	94.0–94.8	94.2–95.4	92.1–92.7	96.8–98.0	88.3–89.1	88.3–88.9	84.1–85.3
C	84.5–86.2	94.3–95.3	93.3–94.6	90.2–92.3	95.6–97.3	89.6–90.9	89.9–91.2	83.2–84.8
E^rns^	84.1–85.3	93.2–94.6	96.5–97.1	92.1–93.1	97.7–98.2	89.4–89.7	89.6–90.5	82.4–8.4
E1	84.4–85.3	93.8–94.7	96.4–97.3	91.3–92.1	97.6–98.5	90.3–91.5	88.4–89.6	81.4–82.1
E2	83.5–84.3	93.5–94.3	95.5–96.5	90.1–91.0	96.6–97.1	87.0–87.5	86.9–87.8	82.0–82.4
P7	79.7–81.6	90.8–93.2	95.2–97.1	91.8–94.2	95.7–98.6	87.4–89.4	88.4–90.3	82.6–85.5
NS2	82.6–83.4	92.9–93.4	96.2–96.6	91.8–92.3	96.9–97.5	89.1–89.6	88.5–89.1	80.2–81.0
NS3	86.4–87.0	94.6–95.1	95.7–96.1	92.8–93.6	97.1–97.4	90.3–91.0	90.8–91.7	85.4–86.1
NS4A	86.2–87.8	94.7–96.3	94.2–95.8	91.0–92.6	97.9–98.9	87.8–89.4	87.8–89.4	82.5–94.1
NS4B	85.8–86.5	92.8–93.6	95.0–95.8	90.1–91.1	96.6–97.8	89.3–90.0	88.4–89.1	83.1–84.1
NS5A	83.7–84.3	93.6–94.0	96.2–96.7	90.7–91.3	97.0–97.3	83.8–84.3	90.6–90.9	82.5–82.9
NS5B	84.7–85.3	94.9–95.3	95.8–96.4	92.5–93.2	97.5–97.8	85.2–85.7	89.5–89.9	83.4–83.7
3’UTR	84.1–85.0	93.8–95.1	94.7–96.0	93.8–95.6	97.3–98.7	83.6–84.4	92.9–94.2	82.6–84.0
Complete	85.0–85.3	94.1–94.4	95.9–96.2	91.9–92.3	97.2–97.5	87.9–88.2	89.6–89.9	83.4–83.7
Amino acid								
N^pro^	91.7–92.9	94.6–95.8	95.8–97.0	94.6–95.8	96.4–97.6	91.7–92.9	92.3–92.9	90.5–91.7
C	92.9–94.9	93.9–97.0	94.9–97.0	91.9–94.9	96.0–98.0	91.9–96.0	92.9–94.9	88.9–90.9
E^rns^	89.0–89.9	96.9–97.8	97.4–98.2	96.9–97.8	98.7–99.6	94.3–95.2	95.6–96.0	90.3–91.2
E1	92.8–93.8	97.4–98.5	96.9–97.9	94.9–95.9	99.5–100.0	96.9–97.9	96.4–97.4	88.7–90.3
E2	90.1–91.2	95.4–96.5	96.2–97.6	94.9–95.7	97.1–98.1	90.9–92.0	91.4–92.5	89.0–90.1
P7	88.4–89.9	95.7–98.6	92.8–95.7	94.2–97.1	95.7–98.6	94.2–95.7	94.2–95.7	91.3–94.2
NS2	89.7–90.6	95.6–96.3	97.4–98.0	95.8–96.3	97.2–98.0	94.7–95.2	93.4–94.1	86.9–87.7
NS3	97.7–98.5	98.7–99.6	98.4–99.1	98.1–98.8	98.1–98.8	97.9–98.7	98.4–99.1	97.8–98.7
NS4A^a^	98.4	100.0	98.4	95.2	100.0	96.8	100.0	95.2
NS4B	95.1–96.3	97.4–98.6	97.7–98.6	98.3–99.4	98.3–98.8	96.5–97.1	97.4–98.6	93.7–94.8
NS5A	87.5–89.0	95.6–96.6	96.2–97.4	93.2–94.8	96.4–97.2	87.1–88.0	93.2–94.0	86.3–87.3
NS5B	92.3–93.2	97.5–98.2	97.6–98.3	97.3–98.0	98.0–98.7	93.0–93.9	96.2–97.2	89.8–90.9

^a^The amino acid homology of NS4A protein of the 8 new isolates between each other was 100%

To explore the genetic variations of the 8 new isolates, the nucleotide sequences of the 5’UTR and the 3’UTR and the amino acid sequences of E2 were fully analyzed by CLC Sequence Viewer 8.0, together with another 23 CSFV isolates from China and other countries.

### Experimental CSFV inoculation of SPF piglets

A total of 10 five-week-old specific pathogen-free (SPF) Bama piglets were obtained from the Experimental Animal Center of the Harbin Veterinary Research Institute, and were randomly divided into three groups (A, B, and C) and maintained in individual biosafety rooms. The piglets in group A (*n* = 3) were inoculated intramuscularly (2 ml) with Shimen strain at a dose of 1 × 10^7.0^ copies. The piglets in group B (*n* = 3) were inoculated with the new CSFV HLJ1 strain with the same method and dose as group A. The piglets in group C (*n* = 4) were mock infected with 2 ml RPMI Medium 1640. Clinical symptoms were observed daily throughout the study, and rectal temperatures were recorded daily before feeding. Blood samples were collected at 0, 7, 14, 21, 28, 31, and 35 days post inoculation (DPI). All remaining pigs were euthanized on 35 DPI. Tissue samples were obtained from the hearts, livers, spleens, lungs, kidney, lymph nodes, tonsils, small intestines, bladders, and stomach for virus detection by RT-PCR, and histopathological or immunohistochemistry (IHC) examination. To determine the level of virulence of the new CSFV HLJ1 strain, the clinical scoring (CS) system was used as described previously [20].

### Histopathological examination and IHC

Tissue samples were subjected to histopathological and IHC examination as previously described [21, 22]. Briefly, tissue samples were fixed in 10% neural buffered formalin for 48 h, embedded in paraffin wax and sectioned. Thin sections with 4-μm thickness stained with hematoxylin and eosin (H–E staining) for histopathological examinations. 4-μm sections were treated with 3% hydrogen peroxide in PBS for 20 min followed by washes in PBS and digestion with 0.05% protease (protease XIV; Sigma) for 5 min at 37 °C. Then washes with PBS, sections were incubated in blocking solutions with 8% skim milk for 40 min at room temperature. After washes with PBS, sections were incubated for 2 h at 4 °C with rabbit antiserum against CSFV (saved by our laboratory) diluted 1:200 in PBS. Then washes with PBS, sections were incubated with anti-rabbit IgG-peroxidase antibody produced in goat (Sigma) diluted 1:400 in PBS for 40 min at room temperature.

## Results

### Virus isolation and genome sequencing

8 new CSFV isolates named HLJ1, SDSG1410, SDLS1410, JSZL1412, HB150309, JL150418, NK150425 and SDZC150601, from 5 provinces (Shandong, Jiangsu, Hebei, Jilin and Heilongjiang) in China, were acquired and sequenced (Table 2). And their complete genome sequences were 12,295, 12,295, 12,296, 12,297, 12,295, 12,295, 12,295 and 12,294 nucleotides in length, respectively.

### Phylogenetic analysis

A total of 8 new CSFV isolates and 44 reference strains, based on the full genome, were used to construct phylogenetic tree (Fig. 1). CSFV isolates were divided into genotype 1, 2 and 3. Genotype 2 isolates were further divided into sub-genotype 2.1, 2.2, and 2.3, and sub-genotype 2.1 isolates could be further subdivided into 2.1a, 2.1b, 2.1c, and 2.1d, which were consistent with the classification results based on E2 and partial NS5B gene [17]. Phylogenetic tree also showed that the 8 new isolates and 3 reference CSFVs (Zj0801, Heb52010 and PC11W) belong to sub-genotype 2.1d. The virulent Shimen strain and its attenuated live vaccine strain HCLV belong to sub-genotype 1.1.

### Analysis of full-length genomic sequences

The complete nucleotide sequences of the 8 new CSFV isolates were compared with 8 representative CSFV isolates, including Shimen, Paderborn, HEBZ, HNSD-20, Zj0801, CSFV39, Alfort and TWN (Table 4). Results indicated that the 8 new isolates shared 97.2–97.5% homology with 2.1d isolate Zj0801, the most closely related strain. Other representative CSFV strains similar to the new isolates were included 2.1a isolate Paderborn and 2.1c isolate HNSD-2012, which shared 94.1–94.4% and 91.9–92.3% homology, respectively. In addition, they shared only 83.4–89.9% homology with 1.1 isolate Shimen, 2.2 isolate CSFV39, 2.3 isolate Alfort or 3.4 isolate TWN. Taken together, these results indicate that all these isolates belong to sub-genotype 2.1d, which was consistent with the result of phylogenetic analysis.

To further examine the genomic variation of the 8 isolates, their genomic characteristics were analyzed in detail. Compared with the representative isolates, the 5’UTRs of the new isolates were the most conserved regions in the genomes, which exhibited 94.4–98.7% homology with 2.1 isolates Paderborn, HEBZ, HNSD-2012 or Zj0801, 93.3–94.1% homology with 2.3 isolate Alfort, 91.6–92.5% homology with 2.2 isolate CSFV39 and 90.3–92.2% homology with 1.1 isolate Shimen or 3.4 isolate TWN. In addition, the amino acid identities of NS3, NS4A and NS4B between the new isolates and representative isolates were higher than other regions of the CSFV genome, and the homologies were 97.7–99.6%, 95.2–100% and 93.7–99.4%, respectively. The detailed identities of the three isolates and eight other representative strain isolates are summarized in Table 4.

### Sequence analysis of UTRs

**Fig. 2:**
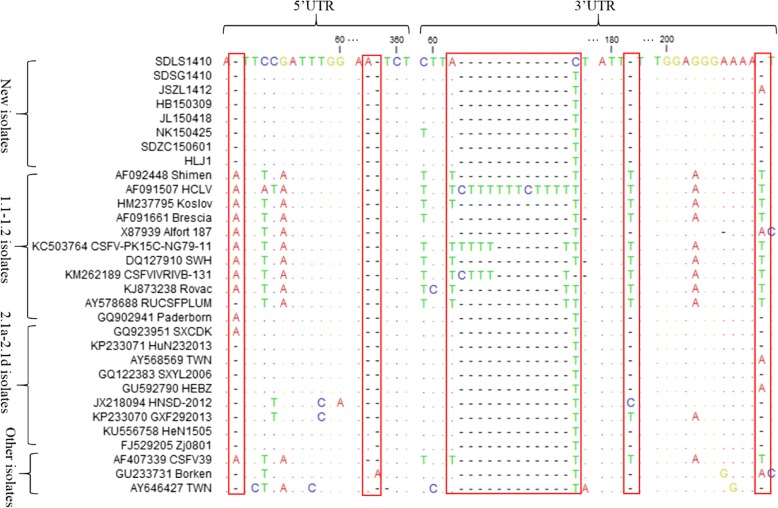
Sequence alignments of 5’UTR and 3’UTR of the 8 new CSFV isolates and 23 reference isolates. Some mutation or deletion regions of these isolates are indicated by red boxes () and described in detail in the text

Compared with sub-genotype 1.1 isolates, the 5’UTRs of the new isolates had a nucleotide A deletion at position 49 (A^49^), which was similar to most of the 2.1 isolates (Fig. 2). In addition, the 5’UTRs of the 6 new isolates (SDSG1410, HB150309, JL150418, NK150425, SDZC150601 and HLJ1) had two continuous nucleotide A deletions at positions 357–358 compared with 2.3 isolate Broken (GU233731), while other 2 new isolates (SDLS1410 and JSZL1412) show only one nucleotide A deletion at these positions (Fig. 2). And most of the CSFV isolates shared the characteristic of the two continuous deletions at these positions (Fig. 2).

Compared with the vaccine strain HCLV, the 3’UTRs of all the 8 new isolates had 12 continuous nucleotide deletions (TTTTTCTTTTTT) at positions 63–74, which were similar to most of the CSFV isolates (Fig. 2). However, among the 23 reference CSFV isolates, the CSFV-PK15C-5NG79–11 (KC503764) strain had 7 nucleotide deletions (CTTTTTT), and the CSFV/IVRI/VB-131 (KM262189) strain had 9 nucleotide deletions (TTCTTTTTT). The other two strains, RUCSFPLUM (AY578688) and Rovac (KJ873238), had 11 nucleotide deletions (TTTTTCTTTTTT) at these positions (Fig. 2). In addition, compared with 1.1 isolates, the 3’UTRs of most 2.1 isolates, including the 8 new isolates, had two discontinuous nucleotide T deletions at positions 182 and 210, respectively (Fig. 2).

### Amino acid analysis of E2

**Fig. 3:**
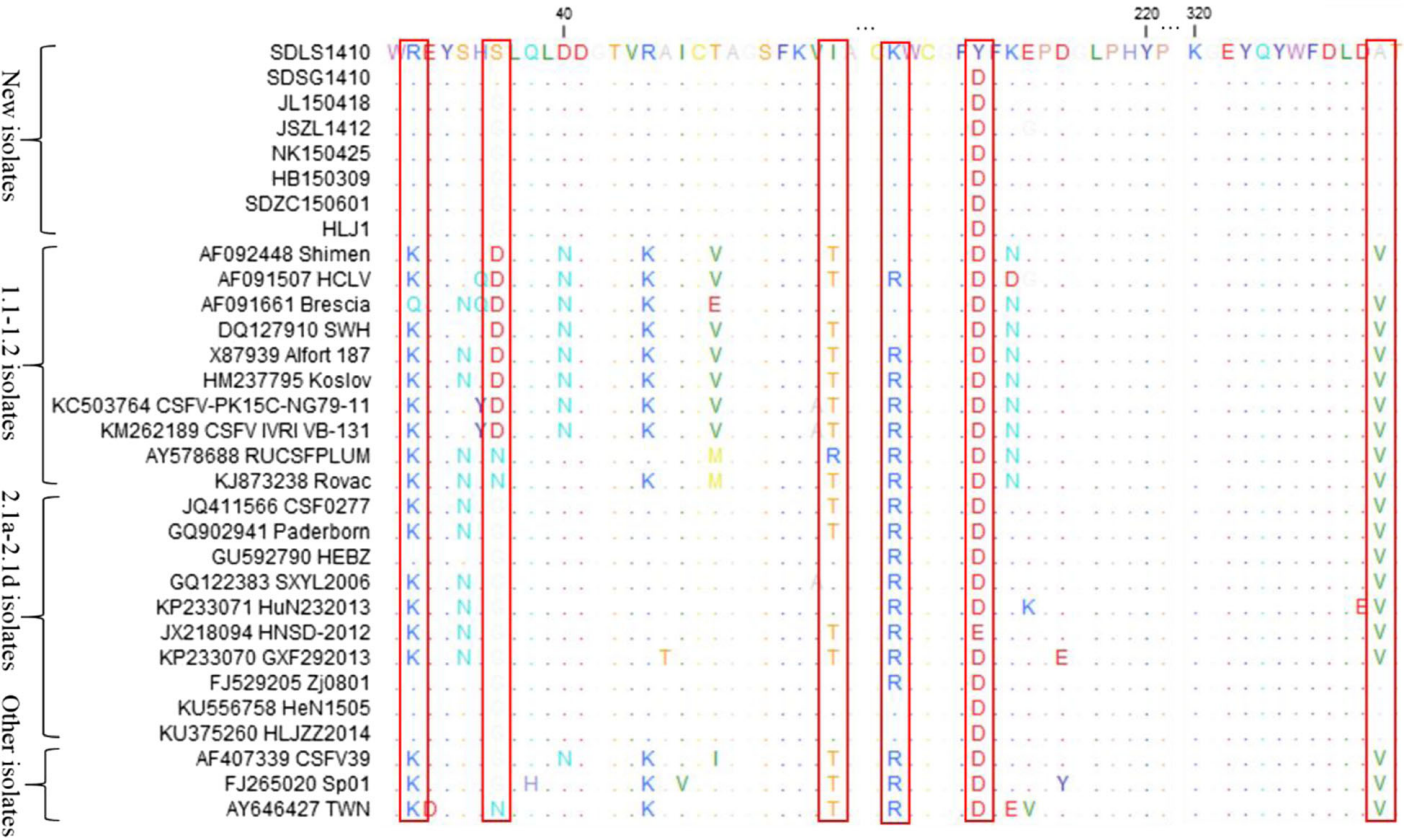
Amino acid sequence alignments of E2 genes of the 8 new CSFV isolates and 23 reference isolates. The special mutation positions of these isolates are indicated by red boxes () and described in detail in the text

The E2 amino acid sequences of the 8 new isolates and 23 reference isolates were compared and analyzed (Fig. 3). And some unique molecular characteristics were found for the new isolates, including the amino acid R at position 31 (R^31^), I^56^, K^205^ and A^331^. And these molecular characteristics were consistent with 2.1d isolates and some 2.1b isolates. In addition, the two new isolates, SDLS1410 and SDSG1410, were S^36^, different from all other isolates. Similarly, at position 210, the SDLS1410 was amino acid Y, and all other sub-genotype isolates were amino acid D (Fig. 3).

### Experimental CSFV inoculation of SPF piglets

**Fig. 4:**
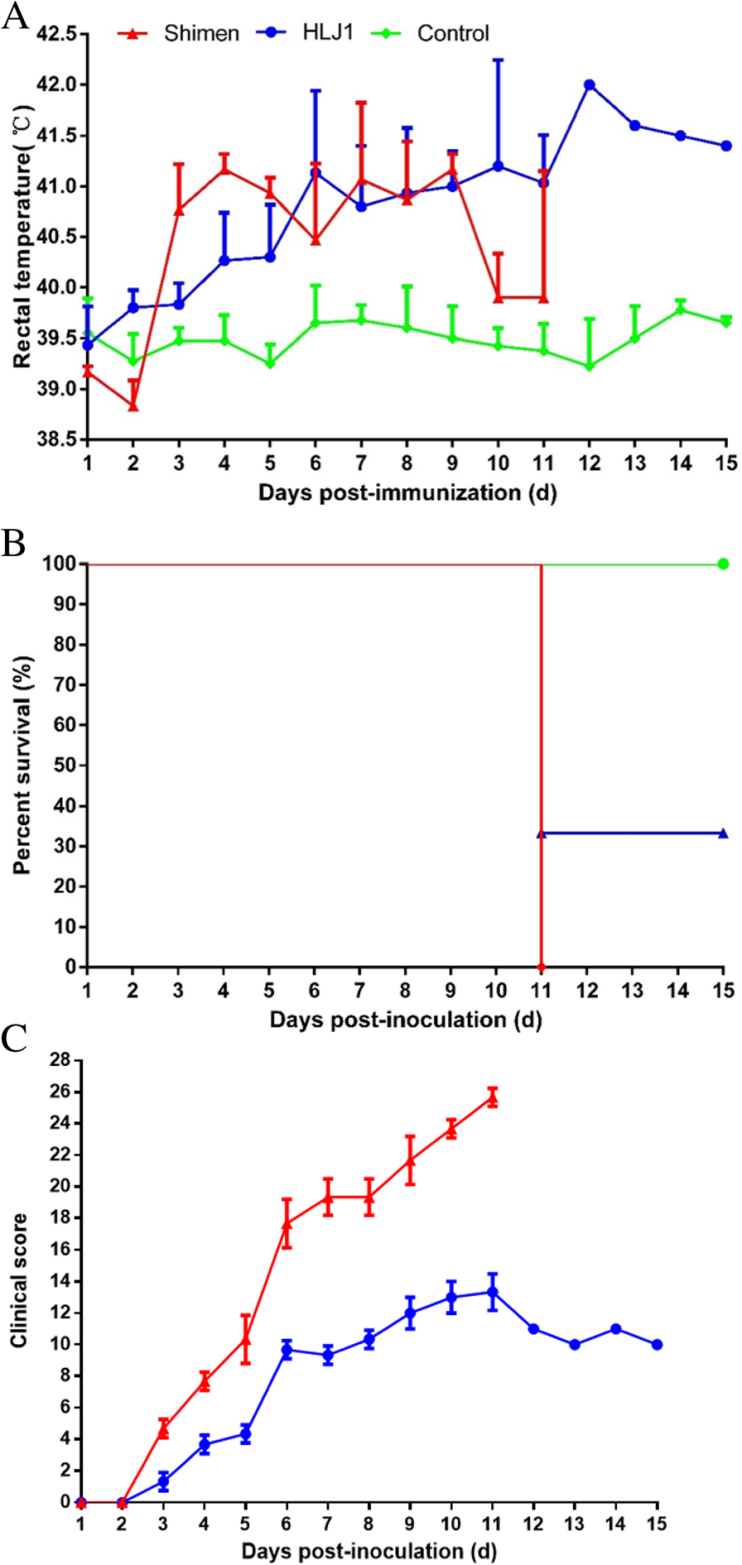
Rectal temperatures (**a**), mortality rates (**b**) and clinical scores (**c**) of piglets inoculated with CSFV Shimen and HLJ1. Mean temperatures ± standard deviations (error bars) are shown (group **a**, *n* = 3; group **b**, *n* = 3 and group **c**, *n* = 4). Rectal temperatures > 40.5 °C were defined as fever

**Fig. 5:**
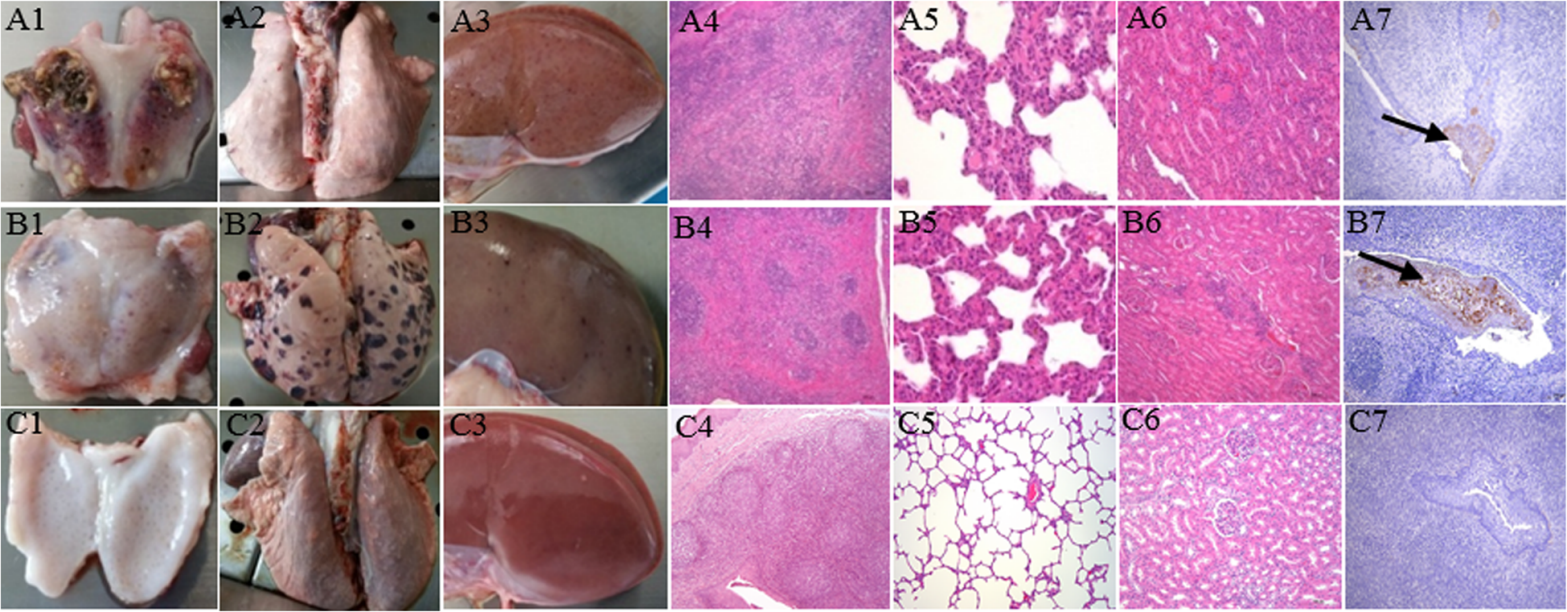
Gross and histological lesions of tonsils, lungs and kidneys of piglets in different groups. Piglets infected with Shimen (**a**1-**a**3) or HLJ1 (**b**1-**b**3) showed obvious necrosis, hemorrhaging or hemorrhagic spots than mock-infected (**c**1-**c**3) piglets. Histopathology of tonsils, lungs and kidneys of Shimen- (**a**4-**a**6), HLJ1-infected (**b**4-**b**6) piglets manifested partial lymphoid follicle atrophy, interstitial pneumonia or lymph histiocytic infiltrates than control (**c**4-**c**6). Viral antigen of CSFV was detected in the tonsils of piglets in Shimen- or HLJ1-infected groups by means of IHC staining (**a**7, **b**7). The tonsils of piglets in control group were negative for CSFV (C7). Original magnification, × 200

The piglets in groups A and B displayed obvious clinical signs, including fever, anorexia and diarrhea, reddening of the conjunctiva and ocular discharge, shivering, and lethargy. In addition, the onset of fever was significantly sooner in group A (Shimen inoculation) than in group B (HLJ1 inoculation) (Fig. 4a). Furthermore, 3/3 piglets in group A died and 2/3 piglets in group B died at 11 DPI (Fig. 4b). The piglets in group C showed no clinical signs throughout the experiment. Based on the CS system, each pig was judged daily after experimental inoculation. The peak CS value of Shimen-infected and HLJ1-infected pigs were determined to be 25–26 and 12–14, respectively, indicating that the HLJ1 strain was moderately virulent (5<peak CS ≤15) (Fig. 4c). The groups A and B piglets showed lesions typical of CSF, such as necrosis in the tonsils, hemorrhaging in the lymph nodes, splenic infarcts and pint hemorrhaging in almost all of the organs (Fig. 5a1-a3 and b1-b3). Histopathology revealed lymphocyte decrease accompanied by partial lymphoid follicle atrophy in tonsils, interstitial pneumonia, and inflammatory lymphohistiocytic infiltrates in the kidney and liver, necrosis of white pulp and lymphocyte depletion in the spleen and varied degrees of hyperemia and hemorrhage in most tissues (Fig. 5a4-a6 and b4-b6 ). In addition, the immunoreactivity was observed in the crypt of tonsil of the piglets in groups A and B (Fig. 5a7 and b7). Virus detection of different tissues and serum samples showed that all samples of piglets in groups A and B were positive, while samples of piglets in group C were negative (data not shown).

## Discussion

CSF is one of the most devastating and highly contagious viral diseases that has caused huge economic losses to pig farms and remains a lingering problem in many parts of the world [23]. Since nationwide vaccinations were implemented, large CSF outbreaks have been rare. However, no regions have been declared free of CSF, and there is still a long way to go to control and ultimately eradicate CSF in China [24]. Four CSFV sub-genotypes, 1.1, 2.1, 2.2, and 2.3, have been identified in mainland China [10]. And a new sub-genotype 2.1c was emerged in China recently [15]. Since late 2014, a new sub-genotype 2.1d isolates were emerged and identified in China [17, 25]. In the present study, we first reported 8 newly emerged CSFV isolates in China in 2014–2015.

The 5’UTR, partial E2 (190 nt), and partial NS5B (409 nt) gene sequences have been widely used for classification of CSFV isolates [26, 27]. Postel et al. reported that the full-length E2 gene sequence provides better resolution for phylogenetic analysis [28]. And identification of sub-genotypes 2.1c and 2.1d was based on the full-length E2 gene sequence [15, 17]. Some labs reported that the whole genome sequence of CSFV could provide more reliable classification criterion [29, 30]. In the present study, the phylogenetic tree was constructed based on the complete genomes. And 8 new isolates were classified into 2.1d sub-genotype (Fig. 1), which was consistent with the results that the phylogenetic tree was constructed based on the full-length E2 gene or partial NS5B gene (data not shown). Interestingly, the 2.1d isolates were located in a relatively concentrated branch close to 2.1b isolates (Fig. 1). The reason may be that sub-genotype 2.1b isolates have become predominant within the last decade in China and the new 2.1d isolates are found to possibly diverge from 2.1b isolates [12–14, 31].

The complete genomic sequences of the 8 new CSFV isolates were compared with other representative CSFV isolates (Table 4). The results indicated that the 8 isolates shared the highest nucleotide homology with Zj0801, the representative strain of sub-genotype 2.1d. And these new isolates also showed high homology with the sub-genotype 2.1b isolate, HEBZ. These results were consistent with the result of phylogenetic analysis. In addition, we also found that the 5’UTRs of the 8 isolates were the most conserved regions in the genomes. And NS3, NS4A and NS4B proteins were more conserved than other regions in the CSFV genome.

The 5’UTR and 3’UTR have been documented to be crucial regulatory elements in the CSFV genome [32]. Sequence alignment showed that the 5’UTRs of the 8 new isolates had the same nucleotide deletions at positions 44 compared with 1.1 isolates (Fig. 2). However, compared with 2.3 isolate Broken, the 6 new isolates (SDSG1410, HB150309, JL150418, NK150425, SDZC150601 and HLJ1) had two continuous nucleotide A deletions at positions 357–358, which was identical to most of the reference CSFV isolates. And other 2 new isolates (SDLS1410 and JSZL1412) show only one nucleotide A deletion at these positions (Fig. 2). Whether the deletions affect the structural characteristics of the 5’UTRs requires experimental confirmation. However, CSFV virulence may vary according to the number and shape of the pseudoknot loop in the secondary structure of 5’UTR and its positional direction in three-dimensional space [29, 33]. Thus, the two groups isolates may have different virulence characteristics.

The 3’UTR is the region with the most variation in the CSFV genome. And the poly-T deletion in the 3’UTR is an important virulence factor and becomes an attenuated-symptom phenotype of CSFV [34]. In the present study, we found that all the 8 new isolates had the poly-T deletion region compared to the vaccine strain HCLV (Fig. 2). We conclude that these isolates were virulent wild CSFV strains with similar virulence. In addition, we also found several previous reported isolates, including CSFV-PK15C-NG79–11, CSFV/IVRI/VB-131, RUCSFPLUM and Rovac, with different deletions in this region, which suggested the differences in the pathogenicity of these strains. Recently, a unique poly-T tract was discovered in the 3’UTR of the CSFV Pinar del Rio strain compared with all other CSFV isolates [35]. And whether this novel insertion affect the pathogenicity of the virus remains unknown. In addition, many studies reported that NS3, NS5A and NS5B of CSFV can interact with 3’UTR to regulate viral RNA synthesis and replication [36–38]. In this study, we also found the 3’UTRs of the 8 new isolates and most sub-genotype 2.1 isolates had two discontinuous nucleotide deletions compared with those of 1.1 isolates (Fig. 2). Whether nucleotide deletions in the 3’UTR affect the interactions with other proteins requires further research.

The E2 protein is the most antigenic protein of CSFV and is involved in virus neutralization [39]. Four antigenic domains, A (86–176 amino acids), B (1–83 amino acids), C (1–110 amino acids), and D (86–110 amino acids), have been mapped on E2 [40]. And domain A has been subdivided into A1, A2, and A3 [40]. In the present study, we found several unique amino acid substitutions for the new isolates on these domains and other regions (Fig. 3). However, the influence of these substitutions on the structure and function of E2 requires further study. In addition, the six cysteines at positions 4, 48, 103, 129, 139, and 167, which were essential for binding by monoclonal antibodies of the four domains, showed no variation in the E2 proteins of these isolates [40]. Furthermore, the potential N-glycosylation sites in the E2 proteins of the 8 isolates were consistent with previous isolates.

The CSFV genotype 1 isolates, such as shimen, showed high pathogenicity, and the Chinese lapinized attenuated vaccine (C-strain) was acquired through the attenuation of the strain, while the genotype 2 or 3 isolates revealed moderate or low virulence [41, 42]. However, Floegel-Niesmann et al. reported that two CSFV isolates, CSF0277 and CSF0849, belonged to sub-genotype 2.1, had been characterized as moderately and highly virulent strains, respectively [43]. In the present study, the pathogenicity of the new HLJ1 isolate which belonged to sub-genotype 2.1d was assessed. Results of animal experiments indicated that the pathogenicity of HLJ1 was less than Shimen (Figs. 4 and Fig. 5), and was similar to HLJZZ2014, a sub-genotype 2.1d isolate with moderate virulence identified recently [44]. In recent years, the C-strain vaccine was widely used to prevent CSFV infection in China. And this vaccine can supply complete protection against the HLJ1 challenging in the lab (data not shown). However, the new sub-genotype 2.1d CSFV isolates could also be epidemic in many immunized pig farms, which was worth of vigilant. The deeper reasons, such as immunization program, vaccine quality, interference by other pathogens and so on, need further study.

## Conclusion

The complete genomes of 8 new CSFV isolates were fully analyzed in the present study. We found that the 8 isolates all belonged to genetic subgroup 2.1d. And some genomic variations or deletions were found in the UTRs and E2. Furthermore, we found that the pathogenicity of the new HLJ1 isolate was less than Shimen. And the sub-genotype 2.1d isolates could be classified into the CSFV strains with moderate virulence. In any case, the epidemic of these isolates should be focus on monitoring and the information of the present study should be considered when establishing control and vaccine strategies for CSF.

## Abbreviations

CSF: Classical swine fever
CSFV: Classical swine fever virus
DPI: Days post inoculation
FBS: Fetal bovine serum
ORF: Open reading frame
SPF: Specific pathogen-free
ST: Swine testicular
UTR: Untranslated region
